# Current and emerging therapies for primary central nervous system lymphoma

**DOI:** 10.1186/s40364-021-00282-z

**Published:** 2021-05-06

**Authors:** Yan Yuan, Tianling Ding, Shu Wang, Hong Chen, Ying Mao, Tong Chen

**Affiliations:** 1 Department of Hematology, Huashan Hospital, Fudan University, 12 Wulumuqi Middle Road, Shanghai, 200040 China; 2 Department of Pathology, Huashan Hospital, Fudan University, 12 Wulumuqi Middle Road, Shanghai, 200040 China; 3 Department of Neurosurgery, Huashan Hospital, Fudan University, 12 Wulumuqi Middle Road, Shanghai, 200040 China

**Keywords:** primary central nervous system lymphoma, diffuse large B-cell lymphoma, targeted therapy

## Abstract

Primary central nervous system (CNS) lymphoma (PCNSL) is a rare type of extranodal lymphoma exclusively involving the CNS at the onset, with diffuse large B-cell lymphoma (DLBCL) as the most common histological subtype. As PCNSL is a malignancy arising in an immune-privileged site, suboptimal delivery of systemic agents into tumor tissues results in poorer outcomes in PCNSL than in non-CNS DLBCLs. Commonly used regimens for PCNSL include high-dose methotrexate-based chemotherapy with rituximab for induction therapy and intensive chemotherapy followed by autologous hematopoietic stem cell transplantation or whole-brain radiotherapy for consolidation therapy. Targeted agents against the B-cell receptor signaling pathway, microenvironment immunomodulation and blood-brain barrier (BBB) permeabilization appear to be promising in treating refractory/relapsed patients. Chimeric antigen receptor-T cells (CAR-T cells) have been shown to penetrate the BBB as a potential tool to manipulate this disease entity while controlling CAR-T cell-related encephalopathy syndrome. Future approaches may stratify patients according to age, performance status, molecular biomarkers and cellular bioinformation. This review summarizes the current therapies and emerging agents in clinical development for PCNSL treatment.

## Introduction

Primary central nervous system (CNS) lymphoma (PCNSL) is an aggressive extranodal lymphoma exclusively involving the brain, spinal cord, cranial nerves, leptomeninges and eyes, with diffuse large B-cell lymphoma (DLBCL) as the most common subtype [1]. The incidence of PCNSL appears to be increasing according to the data from Surveillance, Epidemiology and End Results registries. The highest rates were observed among those age ≥ 65 years. The age-adjusted incidence of PCNSL is 0.16 (0.14-0.18) per 100,000, showing 3-fold increase of extracranial DLBCL between 1985-1997 [2]. CNS infiltration secondary to systemic DLBCLs or lymphomas occurring in immunodeficient patients is excluded from this disease entity [3]. Owing to the limitations of the cranial cavity, patients with PCNSL have intracranial hypertension together with abnormal brain function at presentation and recurrence, showing symptoms of confusion, headache, nausea, vomiting, disorientation, dystaxia, epilepsy, or hemiplegic paralysis with rapid progression [4]. The existence of the blood-brain barrier (BBB) is a major cause of the suboptimal delivery of chemotherapeutic drugs into the CNS, resulting in poorer outcomes in PCNSL than in non-CNS lymphomas [5]. Despite the dramatic therapeutic effects of anti-CD20 antibodies on extracranial mature B cell tumors, the concentration of CD20 antibodies in the cerebrospinal fluid (CSF) is estimated to be 0.14% of that in the peripheral blood [6], raising the point that the overall therapeutic status of PCNSL is lagging in the pre-rituximab (R) era.

A multidisciplinary collaboration is important to control this disease entity. Given the low incidence of PCNSL and the requirement for intraoperative navigation techniques in stereotactic brain biopsy, far fewer randomized clinical trials have been conducted on PCNSL than on non-CNS lymphomas [7]. The current therapeutic strategy can be divided as induction, consolidation and maintenance phases and primarily relies on a high-dose (HD) methotrexate (MTX, M)-based regimen followed by whole-brain radiotherapy (WBRT) [8, 9] or autologous hematopoietic stem cell transplantation (ASCT) [10]. Considering the dose-dependent BBB penetration of cytotoxic agents, balancing the efficiency and side effects of HD chemotherapy remains a major challenge.

**Fig. 1.**
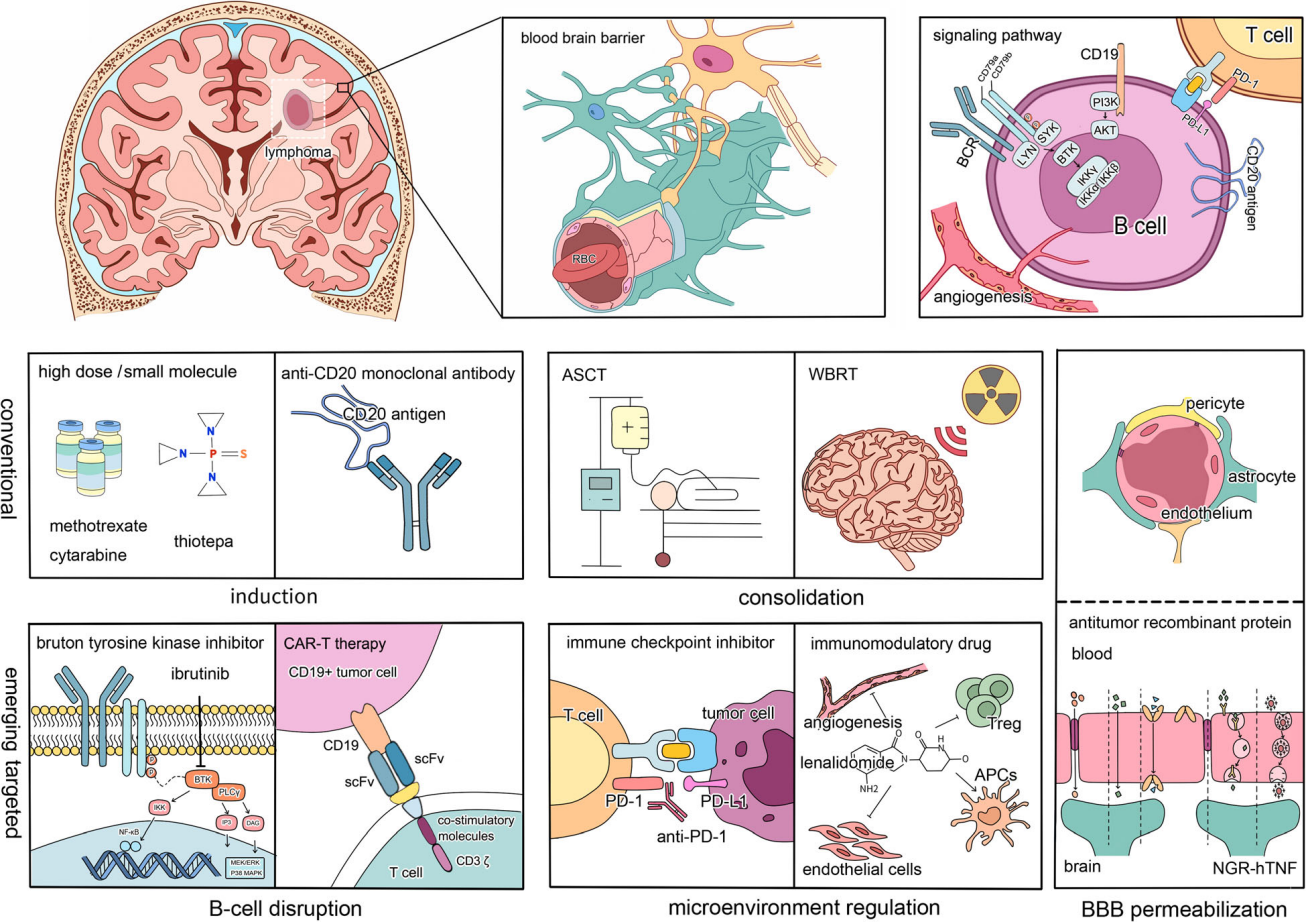
Composition of the BBB and therapeutic strategy for PCNSL. The BBB is a physical barrier formed by endothelial cells connected by tight junctions, the basal lamina, pericytes and astrocyte end-foot processes to protect the brain from attack by circulating microorganisms, chemicals and metabolites. However, it results in relatively low delivery of therapeutic agents to brain tissue from the blood. PCNSL is a type of lymphoma involving only the CNS at the onset, and most cases are pathologically diagnosed as DLBCL. Multi-signaling pathways relating to B-cell development and activation are involved. In addition to conventionally applying high-dose small-molecule chemotherapy during induction and ASCT or WBRT during consolidation, more specific targets in B cells and the BCR signaling pathway, immune microenvironment regulation and BBB permeabilization can be exploited for precision therapy for this disease entity.

Recently, a number of targeted therapies have shown success in treating non-CNS B-cell malignancies [11–13]. Some of these agents showed benefits in preventing disease progression and extending patient survival, providing insight for the future of PCNSL treatment [14, 15]. In this review, we focused on discussing current and emerging therapeutic strategies for this disease entity (Figure 1).

## Diagnosis and pretreatment evaluation

The symptoms of PCNSL are similar to other brain tumors, indicating that the pathological criteria are important in differential diagnosis. As lymphoma cells in newly diagnosed (ND) cases are sensitive to corticosteroids, presteroid biopsy is highly recommended in suspected cases [16]. However, disappearance of the tumor mass after steroid treatment highly indicates its lymphocytic origin, guiding the necessity of interval follow-up and later rebiopsy. In some cases with meningeal infiltration, investigations of the CSF, including cytology, flow cytometry analysis, immunohistochemistry, evaluation of immunoglobulin heavy chain (IgH) rearrangement and detection of other possible genes, are helpful in making an accurate diagnosis [17].

According to the World Health Organization (WHO) classification of tumors of hematopoietic and lymphoid tissue published in 2016 [18], the term primary CNS lymphoma is designated as primary CNS DLBCLs, the majority of which are classified as the non-germinal center B-cell-like (non-GCB) type by cell-of-origin classification with a typical immunohistochemical profile of CD19^+^CD20^+^CD5^-^CD10^-^BCL6^-^ or CD19^+^CD20^+^CD5^-^CD10^-^BCL6^+^IRF4/MUM1^+^. Other B-cell lymphomas with a high tendency to occur within the CNS and pathologically mimic primary CNS DLBCLs, including Burkitt lymphomas, Epstein-Barr virus (EBV)-positive DLBCL, and lymphomatoid granulomatosis, require to be differentiated [19]. As more profound subclassification of DLBCL cases is indicated by analysis of mutated genes [20, 21], detection of EBV-EBER and genetic testing of *MYC* translocation*, BCL2*, *BCL6,* and *IgH* rearrangement, as well as testing for mutations in *MYD88* and *CD79B,* will help to provide more information to aid in interpreting pathologic diagnosis.

To exclude other systemic B-cell malignancies related to CNS infiltration and intraocular involvement, other examinations, including positron emission tomography with computed tomography, bone marrow biopsy/aspirate with flow cytometry analysis, lumbar puncture with CSF detection, fundoscopy and slit lamp microscopy, are necessary for diagnosis and staging [22]. For patients with suspected intraocular lymphoma, optical coherence tomography and vitrectomy are essential for diagnosis, which is aided by immunocytochemistry, flow cytometry, monitoring the interleukin (IL)-10/IL-6 concentration ratio (>1) and clonal detection [23].

## Current therapies for PCNSL

The cutoff for the molecular weight of compounds efficiently penetrating the BBB is 400-600 Da [24]. The majority of the chemotherapeutics currently used to treat PCNSL are small molecules matching this standard and have various BBB penetration efficiencies (Table 1) [14, 15, 25–46].

**Table 1:** Pharmacokinetics of Agents Recommended by NCCN Guideline for PCNSL

	Preferred regimens	Agent ^a^	MW (Da)	Route	Protein binding (%)	CSF (brain)/blood (%)	Reference
Induction	R+MTX(8) R+MTX(8)+ TMZ R+M(3.5)VP(+WBRT in con.) R+MTX(3.5)+TMZ(+WBRT in con.)	MTX(M)	454	iv	46.5-54	2-20	[25, 26]
		rituximab(R)	143857	iv	NA	0.1	[27, 28]
		temozolomide (TMZ)	194	oral	15	20-30	[29–31]
		procarbazine(P)	221	oral	NA	NA	[32]
		vincristine(V)	824	iv	75	undetected	[33]
Consolidation	ASCT with condition regimen BCNU+T TBC HD-AraC(±VP16)	thiotepa(T)	189	iv	NA	95-100	[34]
		carmustine (BCNU)	214	iv	80	20-30	[35]
		Busulfan(B)	246	iv	32	95	[36]
		cyclophosphamide(C)	261	iv	20	50	[37]
		etoposide (VP16)	589	iv	97	0.5-5	[38]
		cytarabine (AraC)	243	iv	13	6-22	[39, 40]
Refractory/Relapse	Retreat with HD-MTX ±R +R+ibrutinib Ibrutinib TMZ Lenalidomide or others	ibrutinib	440	oral	irreversible	1-20(28.7^c^)	[14, 41]
		lenalidomide	259	oral	30	11^p^-20	[42, 43]
		topotecan	421	iv/oral	35	13-68	[44]
		cisplatin	300	iv	90	50	[45]
		pemetrexed	427	iv	81	<5	[46]
		pomalidomide	273	oral	12-44	17-19	[15]

*Abbreviations*: *a* associated conditions collected from the public data sources of drugbank (https://www.drugbank.ca/drugs); *c* corrected for protein binding; *con* consolidation; *CSF* cerebrospinal fluid; *iv* intravenous; *MTX* methotrexate (g/m^2^); *MV* molecular weight; *NA* not available; *NCCN* national comprehensive cancer network; *p* nonhuman primates

Conventional therapy for PCNSL is still staged for induction and consolidation phases. In 2020, the National Comprehensive Cancer Network (NCCN) guidelines recommend systemic therapy for ND patients suitable for or capable of tolerating HD chemotherapy, while for unfit patients, 24-36 Gy of WBRT with a boost to gross disease for a total of 45 Gy is indicated. For ND patients, the first recommendation is HD-MTX at 8 g/m^2^ with rituximab and temozolomide (TMZ) or a reduced dose of 3.5 g/m^2^ MTX with R, vincristine and procarbazine (R-MVP) as well as WBRT [47–49]. When complete remission (CR) or unconfirmed CR (CRu) is achieved, ASCT, low-dose WBRT (23.4 Gy) or continuous monthly MTX administration can be performed as a consolidation therapy, while for patients unable to achieve CR or CRu, WBRT at a dose of 30-36 Gy with a boost to 45 Gy should be performed [9, 49, 50].

### Combined chemoimmunotherapy in the induction phase

**Fig. 2.**
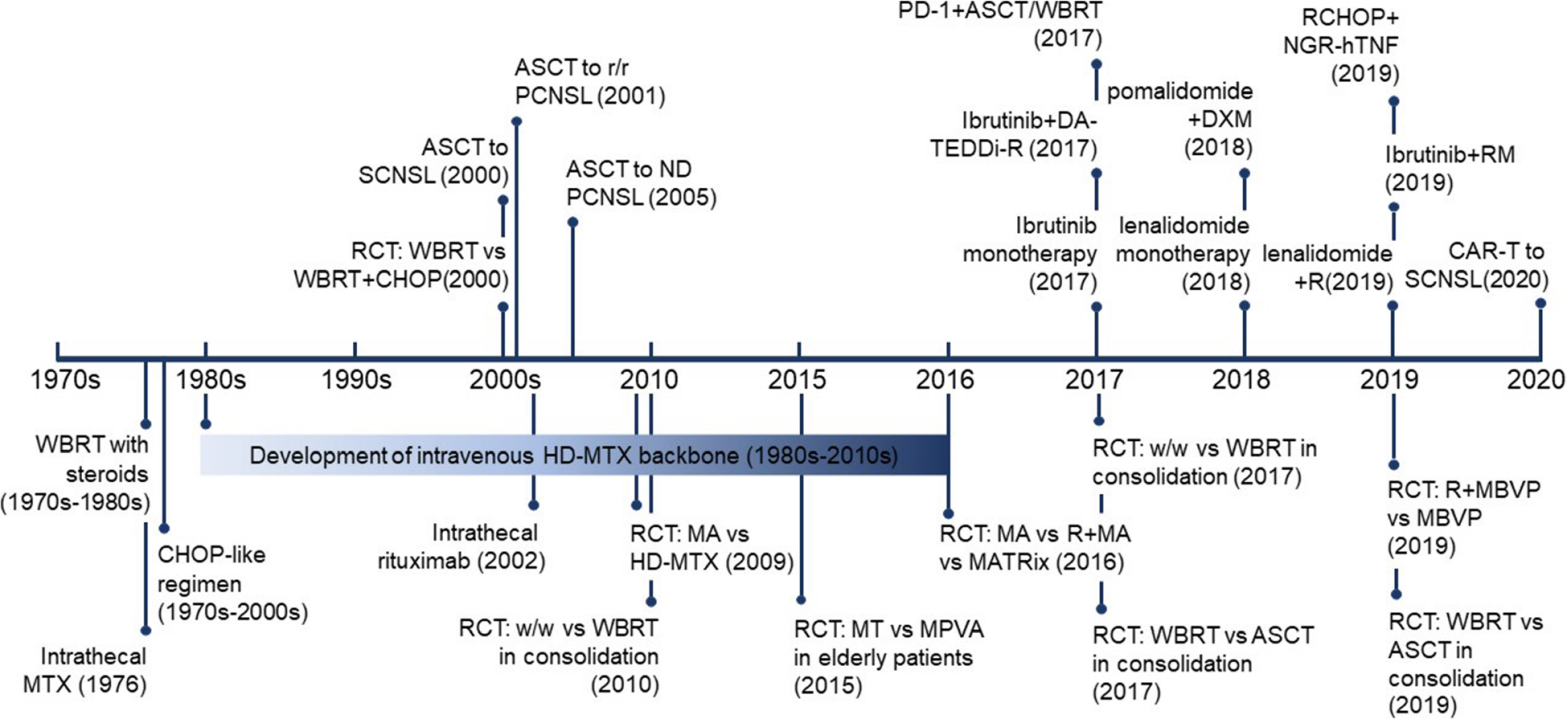
Timeline of the development of targeted chemoimmunotherapy for PCNSL**.** WBRT and the CHOP-like regimen were the major methods used to treat PCNSL in the early 1970s, until high-dose MTX was established as the backbone for chemoimmunotherapy with ASCT or WBRT administered during consolidation. In the past 5 years, more targeted therapies, including rituximab, ibrutinib, and lenalidomide, have been applied to treat ND or r/r patients.

The standard regimen of induction chemotherapy has been modified since the 1970s, with HD-MTX serving as the main backbone (Fig. 2). Additionally, combined agents, including rituximab, cytarabine (AraC, A), temozolomide, procarbazine, vincristine and WBRT, have been evaluated in some randomized clinical trials (Table 2) [51–57], while the regimen of MVP and the addition of rituximab were both described in the early 2000s.

**Table 2:** Randomized Trials for PCNSL

	Year	Diagnosis	N	Age (y)	Regimens (Arms)			Outcome		
								ORR/CR	PFS	OS
Induction	**2000** [51]	PCNSL	53	NA	WBRT(40)	WBRT(40)+CHOP		18% vs 46%	NA	NA
	**2009** [52]	PCNSL, ND	79	18-70	MTX(3.5)	MTX(3.5)+AraC(2)		40% vs 69%	NA	NA
	**2015** [53]	PCNSL, ND	95	≥60	MTX(3.5)+TMZ(150)	MTX(3.5)+Pro(100)+V(1.4)+AraC(3)		71% vs 82%	6.1m vs 9.5m	NA
	**2016** [54]	PCNSL, ND	219	18-70	MTX(3.5)+AraC(2)	MTX(3.5)+AraC(2)+R(375)	MTX(3.5)+AraC(2)+R(375)+T(30) (MATRix)	53% vs 74% vs 87%	42% vs 56% vs 69%	NA
	**2018** [55]	PCNSL, ND	49	14-69	MTX(3.5)+ AraC(1.0)	F(100)+Ten(60)+DXM(40)		40% vs 33%	NA	NA
	**2019** [56]	PCNSL, ND	199	18-70	MTX(3.0)+BCNU(100)+Ten(100)+Pred(60)	R(375, weekly)+MTX(3.0)+BCNU(100)+Ten(100)+Pred(60)		86% vs 86%	(≤60 y) 26.3m vs 59.9m (>60 y) 19.6m vs 14.6m	(≤60 y) 56.7m vs not reached (>60 y) 49.2m vs 34.9m
Consolidation	**2010** [57]	PCNSL, ND	318	55-69	w/w	WBRT(45)		NA	11.9m vs 18.3m	37.1m vs 32.4m
	**2017** [58]	PCNSL, ND	118	18-70	WBRT	ASCT		95% vs 93%	NA	NA
	**2017** [59]	PCNSL, ND	318	55-69	w/w	WBRT(45)		Reduced QoL and lower value of MMSE in WBRT arm		
	**2019** [60]	PCNSL, ND	140	18-60	WBRT	ASCT		NA	63% vs 87%	Cognitive impairment after WBRT

*Abbreviations*: *A* cytarabine (g/m^2^); *BCNU* carmustine (mg/m^2^); *CHOP* cyclophosphamide, doxorubicin, vincristine, prednisone;*CR* complete remission; *DXM* dexamethasone (mg); *EFS* event-free survival; *I* ifosfamide (g/m^2^); *F* fotemustine (mg/m^2^); *M* methotrexate (g/m^2^); *MMSE* Mini Mental State Examinations; *NA* not available; *NCR* no complete remission; *ND* newly diagnosed; *ORR* overall response rate; *OS* overall survival; *PCNSL* primary central nervous system lymphoma; *PFS* progression-free survival; *PR* partial remission; *Pred* prednisone (mg/m^2^); *Pro* procarbazine (mg/m^2^); *QoL* quality of life; *R* rituximab (mg/m^2^); *T* thiotepa (mg/m^2^); *Ten* teniposide (mg/m^2^); *TMZ* temozolomide (mg/m^2^); *WBRT/RT* whole brain radiotherapy (Gy); *w/w* wait and watch; *V* vincristine (mg/m^2^); *VP16* etoposide (mg/m^2^)

Although the role of rituximab in treating PCNSL has been debated for decades, in consideration of its lower delivery in brain tissue, it is incorporated in the most present treatment protocol due to favorable safely spectrum. Apart from some cases of intrathecal injection, rituximab was recently documented to be administered as intravenous infusion at a dose of 375-500 mg/m^2^ weekly or every 2-3 weeks [61, 62]. Two randomized controlled trials (RCTs) designed to compare the outcome of rituximab therapy in PCNSL reported contradicting findings in 2016 and 2019 (Table 2) [54, 56]. One indicated that the addition of rituximab achieved a significantly improved response rate [54], while the other found no clear benefit, although patients <60 years old showed an improved response [56]. The difference in the evaluated endpoint and whether or not WBRT was subsequently applied during consolidation may be the major causes of the opposite conclusions.

The incidence of PCNSL is increased in elderly patients; thus, age is an important factor affecting comorbidities, organ dysfunction and iatrogenic neurotoxicity, all of which are fundamental elements impacting patients’ overall survival (OS) and quality of life (QoL). The outcomes of elderly patients >70 years old, who have a median survival time of 6-7 months, are still challenged, and the fraction of untreated patients >80 years old is dramatically increased [63, 64]. Finding a balance between minimizing toxicity and prolonging survival is complex work indeed.

### ASCT vs WBRT in the consolidation phase

Neurocognitive toxicity is an inevitable side effect of WBRT, and longitudinal neuropsychological assessments need to be performed pre- and post-treatment. Patients undergoing WBRT may exhibit no significant difference in progression-free survival (PFS) or OS at the expense of a decreased global health status, unfavorable physical function and worsened future uncertainty (Table 2) [57, 59, 65]. These issues have raised great interest in evaluating the role of intensive chemotherapy in PCNSL with hematopoietic reconstitution by ASCT [66].

The conditioning regimens used for ASCT have been modified to include drugs with higher delivery into the CNS, and HD thiotepa (T), busulfan (B), cyclophosphamide (C, CTX), AraC, carmustine (BCNU), melphalan and etoposide (VP16, E) are relatively frequently applied (Table 1) [67, 68]. Among these agents, thiotepa exhibits a higher capability to distribute into the CNS following systemic injection and shows an equilibrated concentration between CSF and plasma at 15 minutes post-injection [69]. Compared to other conditioning agents, the regimens including thiotepa show better outcomes and are recommended as the optimal preparation for ASCT (Table 1) [7].

More recently, RCTs evaluating the efficacy and toxicity of first-line consolidation therapies have reported that both intensive therapy with either ASCT or WBRT is effective in PCNSL patients younger than 60 years old, but the patients receiving WBRT exhibit more impacted cognitive disorders (Table 2) [58, 60]. The risks and implications of cognitive impairment after WBRT should be considered when making therapeutic decisions. For the patients younger than 65 years with good performance status, ASCT might be a better choice considering the QoL in an expected long term of survival. However, for the unfit patients ≥65 years or showing symptoms contraindicated to HD chemotherapy, a reduced WBRT will be preferred in consolidation phase.

## Novel targeted therapies and emerging agents

Apart from CD20-targeted rituximab, new findings related to the molecular pathways and pathogenesis of B-cell malignancies have launched the exploration of targeted therapies for PCNSL treatment, most of which are focused on the B cell-related signaling pathway, the immunomodulatory microenvironment, immune checkpoints and BBB permeabilization. These novel targeted agents are creating a new era of precision therapy in the field of PCNSL treatment (Fig. 1, Table 3). The related adverse events (AEs) were listed in Table 4.

**Table 3:** Novel Targeted Therapies for CNS Lymphoma

Author	Year	Diagnosis	N	Study	Age (year)	Regimen	Outcome
BTKi							
Grommes C. et al [70]	2017	PCNSL/SCNSL, r/r	20	phase I	21-85	ibrutinib monotherapy	ORR: 10/13, CR 5, PR 5
Lionakis MS. et al [14]	2017	PCNSL, ND and r/r	18	phase Ib	49-87	ibrutinib + DA-TEDDi-R	CR+CRu 16/18
Soussain C. et al [71]	2019	PCNSL/PVRL, r/r	52	phase II	47-82	ibrutinib monotherapy	ORR: 59%, mean PFS 4.8m, mean OS 19.2m
Grommes C. et al [72]	2019	PCNSL/SCNSL, r/r	15	phase Ib	23-74	ibrutinib + MTX+R	ORR: 12/15
Chen F. et al [73]	2020	PCNSL, ND	11	retrospective	41-68	Ibrutinib+MTX	ORR 81.8%
Narita Y. et al [74]	2021	PCNSL, r/r	44	phase I/II	29-86	tirabrutinib monotherapy	>60% at 320 mg, 100% at 480 mg, and 53% at 480 mg (fasted)
IMids							
Rubenstein JL. et al [42]	2018	PCNSL/SCNSL, r/r	14	phase I	47-79	lenalidomide or lenalidomide +R	ORR: 64%
Tun HW. et al [15]	2018	PCNSL/PVRL, r/r	25	phase I	adults	pomalidomide+DXM	ORR: 48%
Vu K. et al [75]	2019	PCNSL/SCNSL	13	retrospective	70-86	lenalidomide in maintenance	mean PFS: not reached
Ghesquieres H.et al [76]	2019	PCNSL/PVRL, r/r	50	phase II	46-86	lenalidomide+R	ORR: 35.6%
PD-1 antibody							
Nayak L. et al [77]	2017	PCNSL/SCNSL, r/r	5	case report	54-85	nivolumab+R/WBRT	CR 4, PR 1
BBB permeabilization							
Ferreri AJM. et al [78]	2020	PCNSL, r/r	28	phase II	26-78	NGR-hTNF/R-CHOP	ORR: 75%; CR 11, PR 10
CAR-T							
Tu S. et al [79]	2019	PCNSL, r/r	1	case report	67	CD19-CD70 dual CART	CR
Abbasi A. et al [80]	2020	SCNSL, r/r	2	retrospective	NA	axicabtagene ciloleucel	CR 2
Abramson JS. et al [81]	2020	SCNSL, r/r	7	prospective	NA	lisocabtagene maraleucel	CR 3 (of 6 evaluated)

*Abbreviations*: *CART* chimeric antigen receptor T cells; *CNS* central nervous system; *CR* complete remission; *CRu* complete remission unconfirmed; *DA-TEDDi-R* etoposide, temozolomide, liposomal doxorubicin, dexamethasone, intrathecal cytarabinem; *DXM* dexamethasone; *MTX* methotrexate; *NA* not available; *ND* newly diagnosed; *NGR-hTNF* tumor necrosis factor-α coupled with CNGRCG peptide; *ORR* overall response rate, *OS* overall survival, *PCNSL* primary central nervous system lymphoma; *PFS* progression-free survival; *PR* partial remission, *PVRL* primary vitreoretinal lymphoma; *R* rituximab; *r/r* refractory/relapse; *SCNSL* secondary central nervous system lymphoma; *WBRT* whole brain radiotherapy

**Table 4:** Major AEs of Targeted Therapies for CNS Lymphoma

Targeted therapy	Adverse events		
	Events	Any grade	Grade≥3
ibrutinib [14, 70–72]	neutropenia	25%(5/20)~40%(6/15),	3.8%(2/52)~ 83%(15/18)
	anemia	70%(14/20)~100%(15/15)	5%(1/20)~20%(3/15)
	thrombocytopenia	70%(14/20)	10%(2/20)~72%(13/18)
	febrile neutropenia	5%(1/25)	1.9%(1/52)~61%(11/18)
	increased creatinine	27%(4/15)~30%(6/20)	27%(4/15)
	increased ALT	3.8%(2/52)~80%(12/15)	7%(1/15)~10%(2/20)
	diarrhea	3.8%(2/52)~25%(5/20)	7%(1/15)~11%(2/18)
	prolonged APTT	20%(3/15)~30%(6/20)	10%(2/20)
	infection	9.6%(5/52)~27%(4/15)	5.8%(3/52)~78%(14/18)
	atrial fibrillation	3.8%(2/52)	1.9%(1/52)
lenalidomide [42, 76]	neutropenia		21.4%(3/14)~40%(20/50)
	thrombocytopenia		10%(5/50)
	anemia		4%(2/50)
	infection		8%(4/50)~21.4%(3/14)
Pomalidomide [15]	neutropenia	100%(25/25)	20%(5/25)
	thrombocytopenia	44%(11/25)	8%(2/25)
	anemia	80%(20/25)	8%(2/25)
	thromboembolism	8%(2/25)	
	infection	44%(11/25)	16%(4/25)
	fatigue	40%(10/25)	8%(2/25)
	dyspnea, hypoxia and/or respiratory failure	16%(4/25)	16%(4/25)
nivolumab [77]	pruritus	20%(1/5)	
	fatigue	20%(1/5)	
	renal insufficiency	20%(1/5)	20%(1/5)
NGR-hTNF [78]	neutropenia	89%(25/28)	85%(24/28)
	thrombocytopenia	85%(24/28)	61%(17/28)
	anemia	85%(24/28)	21%(6/28)
	febrile neutropenia	14%(4/28)	14%(4/28)
	infection	14%(4/28)	14%(4/28)
	deep vein thrombosis	7%(2/28)	7%(2/28)
	infusion reaction	32%(9/28)	
	hepatotoxicity	50%(14/28)	18%(5/28)

*Abbreviations*: *ALT* alanine aminotransferase, *APTT* activated partial thromboplastin time

### Bruton tyrosine kinase inhibitor (BTKi)

The pathogenesis of the non-GCB type of PCNSL involves a B cell-activating process after B cells migrate from the dark zone to the light zone, which leads to activation of B-cell antigen receptor (BCR) and induces NF-κB-mediated cell proliferation [82]. BTK is a factor in the downstream signaling pathway, serving as an inhibitor of Fas-induced apoptosis in B lymphocytes [83] and playing a role in B cell development independent from the myeloid signaling pathway [84]. A single amino acid substitution or multiple mutations in BTK mediate the phenotype of X-linked immunodeficiency in humans and mice, displaying defects in cyclins and cycle entry blockade in B cells [85].

Ibrutinib, the first-in-class BTKi, exerts a specific function to disrupt BCR signaling. As a unique small-molecule inhibitor selectively binding to BTK in the context of a complex proteome to inhibit B cell activation [86], ibrutinib has succeeded in treating some mature B-cell neoplasms, including chronic lymphocytic leukemia (CLL) [11], mantle cell lymphoma [12], and Waldenström macroglobulinemia [87], as well as some non-GCB-type DLBCLs [88]. Ibrutinib has been reported to significantly block the anti-IgM-induced cellular survival and DNA replication of CLL cells [89]. The second generation of BTKis including zanubrutinib (BGB-3111), acalabrutinib (ACP-196) and orelabrutinib (ICP-022) show more selectivity for the BTK pathway and less off-target effects on EGFR, TEC, IL-2 tyrosine kinase, and other kinases [90], increasing the efficacy and safety in B-cell malignancy treatment [91, 92].

To date the potential relevant of ibrutinib to PCNSL, the pharmacokinetics of ibrutinib and its primary metabolite, PCI-45227 was investigated either in plasma or in CSF. Interestingly, free ibrutinib achieved significant CNS penetration at doses from 560 mg to 840 mg, with a 28.7% CSF/plasma ratio after correction for protein binding (Table 1) [14]. Another preclinical study also provided evidence of the BBB-permeabilizing efficiency of ibrutinib, showing that ibrutinib rapidly crossed the BBB in 0.29 h (0.2-0.32 h) [41].

The first successful therapeutic application of a BTKi in PCNSL was published in 2017. Eighteen patients with relapsed/refractory (r/r) PCNSL were treated with 560-840 mg of ibrutinib combined with a chemotherapy regimen, most of which were a DA-TEDDi-R regimen including temozolomide, etoposide, liposomal doxorubicin, dexamethasone, ibrutinib, rituximab, pegfigrastim and intrathecal injection of AraC. Twelve of 14 evaluated patients achieved CR or CRu, and 57% (8/14) of the patients continued to be progression-free at a median time of 15.5 months of follow-up. The median PFS time was 15.3 months, while median OS endpoint was not reached [14]. Two other designed ibrutinib dose-escalation studies at a daily dose from 560 mg to 840 mg showed 12/15 (80%) and 10/13 (77%) of patients achieved a response, including eight and five CRs, respectively [70, 72]. Another phase I/II study enrolled 44 r/r PCNSL patients with orally dose escalating tirabrutinib, a second generation BTKi in Japan. Independent review committee assessed overall response rate (ORR) of 64% (5 CR/CRu) at 320 mg, 100% (4 CR/CRu) at 480 mg, and 53% (6 CR/CRu) at 480 mg under fasted condition [74]. However, ibrutinib monotherapy produced shorter PFS than combined therapy [14, 71, 72, 93].

The optimal combination of chemotherapy with BTKis has not yet been developed. In a study on DA-TEDDi-R, the antifolate agents, including methotrexate, did not exhibit synergistic action with ibrutinib in a cytotoxicity assay performed with TMD8 and OCI-Ly10 lymphoma cells, while the DNA-damaging agents did. Instead, an antagonist representative DBSumNeg metric value indicated the antagonistic relationship between ibrutinib and multiple antifolates [14]. However, another ibrutinib-based regimen combined with HD-MTX did show an increased 80% (12/15) response in r/r patients [72]. Recently, a real-world experience of combining ibrutinib with HD-MTX in treating ND PCNSL patients reported 81.8% of ORR with mind tolerable anemia, hypoalbuminemia and hypokalemia, indicating a potential role of BTKi in the frontline treatment [73]. A couple of BTKi-based trials evaluating ibrutinib, acalabritinb or orelabrutinib in combination with the agents HD-MTX, R-MVP, MRE, lenalidomide or an anti-programmed death (PD)-1 antibody are currently ongoing (Table 5). The standard regimen and the functions of various BTKis remain to be determined.

**Table 5:** Registered BTKi Trials of CNS Lymphoma on ClinicaTrial.gov

Trial	N	Recruited condition	Study	Intervention regimen	Location
NCT03581942	45	PCNSL, r/r	Ib/II	dose escalation ibrutinib+copanlisib	USA
NCT03770416	40	PCNSL/SCNSL, r/r	II	ibrutinib+nivolumab	USA
NCT02315326	63	PCNSL/SCNSL, r/r	I/II	dose escalation ibrutinib+HD-MTX+rituximab	USA
NCT02623010	30	PCNSL after CR/PR	II	ibrutinib in maintenance	Israel
NCT03703167	40	PCNSL/SCNSL, r/r	Ib	dose escalation ibrutinib+lenalidomide+rituximab	USA
NCT04421560	37	PCNSL, relapse	Ib/II	dose escalation ibrutinib+ pembrolizumab +rituximab	USA
NCT04129710	120	PCNSL, r/r	II, RCT	ibrutinib+MRE vs lenalidomide+MRE	China
NCT04066920	30	PCNSL, r/r, transplant ineligible	II	IBER in induction +ibrutinib in maintenance	Korea
NCT02203526	52	PCNSL	I	TEDDi-R+isavuconazole	USA
NCT03964090	32	SCNSL	II	TEDDi-R+isavuconazole	USA
NCT04446962	128	PCNSL, ND	Ib/II, randomized	dose escalation R-MVP+ibrutinib vs R-MVP+lenalidomide	France
NCT04462328	21	PCNSL/SCNSL	I	dose escalation acalabritinb+durvalumab	USA
NCT04438044	39	PCNSL/SCNSL, r/r	II	orelabrutinib	China

*Abbreviations*: *BTKi* bruton tyrosine kinase inhibitor;*CNS* central nervous system; *CR* complete remission; *DA-TEDDi-R* etoposide, temozolomide, liposomal doxorubicin, dexamethasone, intrathecal cytarabine; *HD-MTX* high dose methotrexate; *IBER* ibrutinib, rituximab, ifosfamide and etoposide; *MRE* methotrexate, rituximab, and etoposide; *ND* newly diagnosed; *PCNSL* primary central nervous system lymphoma; *PR* partial remission; *RCT* randomized control trial; *R-MVP* rituximab, methotrexate, procarbazine and vincristine; *r/r* refractory/relapse; *SCNSL* secondary central nervous system lymphoma; *TEDDi-R* temozolomide, etoposide, doxil, dexamethasone, ibrutinib and rituximab

Tyrosine kinase signaling is also required for the innate immune system and T cell function. BTK-deficient macrophages lack IL-10 production, which is stimulated by Toll-like receptor activation, and nuclear localization of NF-κB and AP-1 [94]. *Aspergillus fumigatus*-stimulating phagocytosis was indicated to be mediated by BTK signaling and was inhibited by BTK-siRNA or ibrutinib [95]. BTKis also inhibit IL-2-inducible T-cell kinase (ITK) signaling in the CD4^+^ T helper 2 (Th2) cell population, which causes failures in protection against pathogens [96]. As every coin has two sides, the impact of BTKis on cell cycling results in hampered anti-infectious functions of B cells. This AE occurred in 56% of patients treated with ibrutinib monotherapy and 52% of patients treated with combined therapy [97], with pulmonary/CNS aspergillosis being more frequent in the heavily treated group[14, 72, 98].

The relationship between ibrutinib resistance and genetic mutations in CNS lymphoma is still unclarified. Two studies reported genomic findings related to the response to ibrutinib. In an open-label, nonrandomized, dose-escalation ibrutinib monotherapy trial with whole-exon sequencing detection, twenty-six genes were found to be recurrently mutated in PCNSL, and 23 additional genes were attributed to aberrant somatic hypermutation (aSHM). Among the genes, with a high frequency of *MYD88* and *CD79B* mutation, *PIM1, BTG2, PRDM1, TOX*, and *IRF4* were scored as both recurrent mutations and targets of aSHM. In 4/20 patients documented as incompletely response, one patient had a mutation in the coiled-coil domain of *CARD11* (R179Q), while the other three had a mutation in *CARD11* (R337Q) or inactivating lesions in *TNFAIP3* (deletion or frameshift mutation) [70]. In another phase Ib trial of rituximab + 3.5 g/m^2^ MTX + ibrutinib, the patients were screened with *MYD88, CD79B, CARD11, TNFAIP3* and *PLCG2*. Two patients had progressive disease (PD), and one had stable disease (SD) in this study, all of which had a histological GCB type. One PD patient showed a wild-type genotype, and the other had a *CARD11* mutation (T128M/K252E). The SD patient had a *PLCG2* mutation (R268W). Interestingly, the patients with mutations in *CARD11* exhibited diverse ibrutinib responses (CR, partial response [PR], or PD) [72]. Thus, the specific BTKi resistance gene and whether combined therapy is able to overcome mutation-driven resistance need further investigation.

### Immunomodulatory drugs (iMiDs)

IMiDs are able to increase natural killer (NK) cell cytotoxicity, augment T cell proliferation, increase production of IL-2 and interferon (INF)-γ, and modulate IL-12 expression. The synergistical enhancement of NK cell- or monocyte-mediated antibody-dependent cellular cytotoxicity has been seen in combining iMiDs and rituximab-treated non-Hodgkin’s lymphoma, supporting the use of iMiD-containing treatments in r/r B-cell lymphomas [99].

Lenalidomide, a second-generation iMiD, was demonstrated to increase INF-β production, downregulate *IRF4-SPIB* expression and reduce *CARD11*-transactivating prosurvival *NF-κB* signaling to synergize with BTKi to kill activated B-cell type DLBCL cells [100]. The BBB penetration efficiencies of lenalidomide and pomalidomide (Pom) were quantified in two studies. Based on 23 matched plasma-CSF samples, the CSF/plasma ratio of lenalidomide ranged from 0%-49%, with dose-dependent CSF penetration efficiencies of 10%, 20.4%, and 25.5% at daily doses of 10 mg, 15 mg, and 20 mg, respectively [42]. Pom, a third generation iMiD, exhibits a similar capability for BBB penetration. Approximately 4-4.6 hours after oral administration, the concentration of unbound Pom in the blood and CSF reached 1,100 ± 82 ng/mL and 430 ± 63 ng/mL in a rat model, with an unbound AUC_brain_ to AUC_blood_ ratio of 39 ± 3% [101]. In a r/r patient treated with a daily dose of 3 mg of Pom, the CSF/blood ratios on day 1 and day 14 were 17% and 19%, respectively [15].

Lenalidomide monotherapy or lenalidomide+rituximab (R2) combined therapy was applied to treat r/r central nervous system lymphomas (CNSLs) during induction or maintenance [42, 75, 76]. Considering BBB penetration efficiency and dose-related AEs, lenalidomide in the R2 regimen was given at a dose of 15-25 mg/day for 21 days for a 28-day cycle [42, 76], while a reduced dose of 5-10 mg was reported for maintenance therapy[42, 75]. Interestingly, the response rate of CNSL to iMiDs was associated with the tumor location, while the underlying mechanism has not been identified. According to intention-to-treat data, the ORR of lenalidomide monotherapy was approximately 64%, with a higher response in parenchymal lesions than in leptomeningeal disease (60% vs 33%) [42]. Another prospective phase II study conducting R2 induction reported 12 CRs and 10 PRs in 35 cases of PCNSL, with an ORR of 64.7%. However, for patients with vitreoretinal lymphoma, the ocular CR rate was only 35% [76]. The ORR for Pom treatment was found to be 48% in r/r PCNSL and primary vitreoretinal lymphoma (PVRL) patients [15]. Exciting evidence shows that lenalidomide during maintenance is beneficial for r/r patients. The PFS of a cohort of recurrent patients in CR2-CR5 with lenalidomide maintenance was 6 times longer than that in a retrospective cohort of CR1 patients treated with conventional therapy [42].

The grade 3/4 iMiD-related AEs in CNSL are mainly cytopenia, followed by nonhematologic lung infection, sepsis, fatigue, syncope, dyspnea, hypoxia, respiratory failure and rash [42, 76]. No patient had febrile neutropenia >7 days in the POM-DXM study [15]. However, the information on iMiDs in PCNSL is still inadequate. Although deletion of *Cereblon (CRBN)*, a target of iMiDs linked with interferon regulatory factor 4 (*IRF4*), has been associated with iMiD resistance in myeloma, the mechanism of iMiD resistance in PCNSL has not yet been clarified [102].

### Checkpoint inhibitors

More recently, the expression of checkpoint receptors and their ligands has attracted increasing interest in tumor immune privilege. Some hematologic malignancies, especially Hodgkin’s lymphoma, has been demonstrated to overexpress programmed cell death protein (PD)-1 ligand, and anti-PD-1 antibodies block the ability of tumor cells to escape from immune surveillance [103].

PD-1 (CD279), encoded by the gene *PDCD1* on chromosome 2q37.3, was first discovered in 1992 [104], and its ligands PD-L1 (CD274) and PD-L2 (CD273) were found to be harbored on 9p24.1 [105–107]. Recurrent genetic features, including gene mutations, copy number alterations (CNAs) and chromosomal rearrangements, are frequently seen in PCNSL [108]. Similar to primary mediastinal large B-cell lymphoma, PCNSL cases have a relatively high frequency of 9p24.1/*PD-L1/PD-L2* CNAs, whose structural bases cause overexpression of PD-L1/PD-L2 and related immune evasion. It has been estimated that 67% of EBV^-^ PCNSL cases exhibit 9p24.1/*PD-L1*/*PD-L2* copy gain and CNA-associated increased expression. EBV triggering PD-1 upregulation is an additional mechanism in EBV^+^ PCNSL. These indicate the potential of checkpoint inhibitors for PCNSL treatment [108].

A retrospective study investigated whether PD-L1 and PD-L2 are expressed at lower levels on PCNSL tumor cells than on peritumoral macrophages [109]. High tumoral PD-L1 signaling and low PD-1^+^CD8^+^ T cell infiltration were indicated to be associated with an inferior prognosis in PCNSL [110, 111]. A pilot study showed that nivolumab (an anti-PD-1 antibody) produced superior clinical and radiographic responses in five r/r PCNSL or secondary CNS lymphoma (SCNSL) patients. The regimens used in the study were not identical. Only one patient was receiving corticosteroid treatment at the time of nivolumab administration, while the others were administered nivolumab immediately after radiotherapy or ASCT [77]. Another r/r PCNSL patient was reported to be successfully treated with nivolumab during maintenance. Even though the patient’s archival tumor tissue showed no expression of PD-L1/PD-L2 and no amplification of the PD-L1 locus on 9p24.1, the patient achieved durable remission after a 3rd round of ASCT with nivolumab maintenance [112].

### BBB permeabilization

Another potential approach is to permeabilize the BBB by targeting aminopeptidase N (CD13), a membrane-bound metalloproteinase upregulated in tumor angiogenesis [113]. By fusing the N terminus of human tumor necrosis factor (TNF)-α with CNGRCG, a protein called NGR-hTNF was generated and was able to recognize CD13 and increase leakage from the vasculature to tumor tissue, resulting in an enhancement in antitumor effects [114].

CD13 is widely expressed on tumor vessels, epithelia, kidney tissue and myeloid cells. However, only the anti-CD13 VM15 isoform can recognize NGR-TNF, which is highly dependent on the NGR domain [115]. Synergistic tumor vessel damage and tumor-eradicating function were observed after the coadministration of NGR-TNF with chemotherapeutics (doxorubicin, cisplatin, paclitaxel and gemcitabine) or other antitumor cytokines (INF-γ and endothelial-monocyte activating polypeptide II) [116–118].

A phase II trial showed that the combination of low-dose NGR-hTNF with a standard R-CHOP regimen produced a remarkable response in r/r PCNSL patients [119, 120]. NGR-hTNF was administered from cycles 2-6 at a dose of 0.8 μg/m^2^ 2 hours before CHOP21 in a 1-hour infusion. NGR-hTNF/R-CHOP was well tolerated, and no unexpected toxicities, toxicity-related course interruptions or dose reductions occurred. Among all cycles, there were sixteen episodes of serious AEs, including seizures, deep venous thrombosis, infection, syncope, constipation, febrile neutropenia and left ventricular function reduction. The ORR was 75% (11 CRs and 10 PRs among 28 patients), which has restored interest in using R-CHOP to treat CNSL [78].

### Chimeric antigen receptor (CAR) T cells

CAR-T cells have been used to successfully treat B-cell leukemia and lymphoma, primarily by integrating domains for CD19 recognition and T cell activation to eradicate CD19-expressing malignant cells [121]. However, their potential in PCNSL has not yet been thoroughly explored, primarily because of treatment-related AEs (TRAEs), especially CAR-T cell-related encephalopathy syndrome (CRES).

Cytokine release syndrome (CRS) and CRES are the major toxicities of CAR-T cell therapy, but the underlying mechanisms are different. The severity of CRES may be associated with the number of activated T cells and levels of related cytokines in the CNS. In a rhesus macaque model injected with CD20 CAR-T cells and non-CAR-T cells, widespread intraparenchymal T cell infiltration comprising both CAR-T cells and non-CAR-T cells was observed in all brain regions. However, the expression of the integrin VLA4 in CAR-T cells was 1.5-2 times of that in non-CAR-T cells, indicating that the CAR-T cells were more capable of homing into the brain. The levels of multiple proinflammatory cytokines, including IL-6, IL-2, GM-CSF, and VEGF, were elevated in the CSF by 478-, 10-, 8.2- and 6.3-fold, respectively [122]. These asymmetric increases in cerebral proinflammatory cytokine levels were also detected in an acute lymphocytic leukemia patient given CD19 CAR-T cell treatment [123].

IL-6, the major pathogenic macrophage/monocyte-released cytokine, functions as a proinflammatory or anti-inflammatory factor via soluble IL-6R-mediated “trans-signaling” or membrane-bound IL-6R-mediated “cis-signaling”, respectively, and trans-signaling results in a high level of IL-6 and affects almost all types of cells [124]. Thus, tocilizumab, a humanized anti-IL-6-receptor (IL-6R) monoclonal antibody (mAb), has been applied to block IL-6 receptor to manage hypotension, hypoxia and widespread organ dysfunction [125]. In an SGM3 mouse model established with patient-derived CD19^+^CD44v6^+^ ALL-CM cells, the mice were coinjected with nHuSGM3 T cells transduced with the CD44v6.28z CAR or CD19.28z CAR. Tocilizumab or anakinra (an IL-1 receptor antagonist) was administered just before CAR-T cell injection to prevent TRAEs. Interestingly, the mice treated with tocilizumab showed a sudden lethal neurologic syndrome on days 27-33, while those treated with anakinra did not, indicating that IL-1 signaling activation is the key mechanism in CAR-T cell-related neurotoxicity [126].

Recently, a high-throughput single-cell RNA-sequencing study found that in the human brain, there is a small population coexpressing the B cell marker CD19 and the mural cell marker CD248. This population does not express CD79a or αSMA, highlighting that the cells are indeed pericytes coexpressing CD19. Interestingly, pericytes obtained from human lung tissues did not show CD19 staining. In a B cell-independent NSG mouse model, this specialized cerebral expression pattern caused damage to mural cells through off-tumor targeting by CD19 CAR-T cells and resulted in increased BBB permeabilization, as measured by Evans blue dye diffusion, during treatment with mCD19-28z CAR-T cells [127]. Additional supportive evidence for CD19 coexpression in mural cells is, in an experiment staining infiltrating CD3^+^ T cells in the CNS, a cerebral perivascular positive signal was seen in a patient with CD19-CAR-T cell-related CRES, indicating that the brain perivascular tissues are also targeted by CAR-T cells [128]. However, T cell therapies specific for other B cell antigens, including CD20 and CD22, also exhibit treatment-related neurotoxicity in either humans or animals, highlighting the complicated underlying mechanism of immune effector cell-associated neurotoxicity syndrome (ICANS) [122, 129].

There have been some reports of CNS lymphoma showing promising outcomes with CAR-T cell therapy. A 67-year-old patient with PCNSL was treated with CD19 CAR-T cells and CD70 CAR-T cells. The patient had symptomatic improvement and obtained CR after one month. CART19 and CART70 cells were detectable in 10 months. No CRS and CRES occurred [79]. FDA-approved axicabtagene ciloleucel and lisocabtagene maraleucel (liso-cel) showed response to SCNSL [80, 81]. Compared to the patients without CNS involvement, the SCNSL patients had a decreased incidence of CRS and an increased incidence of grade 3-4 ICANS with liso-cel treatment [81]. Considering the function of IL-6 in CRS and CRES, some studies have reported preventing TRAEs by silencing IL-6 signaling. Adding a short hairpin RNA expression cassette specific for IL-6 to a conventional CAR vector significantly reduced monocyte-released IL-6 levels and did not cause ICANS in a pilot study including 3 CNSL patients [130, 131].

## Future perspectives

In the rituximab era, the overall therapeutic status of PCNSL patients lags far behind that of non-CNS DLBCL patients, resulting from the existence of a physiological barrier and unique pathology. The activated B-cell subtype with mutations in MYD88 and CD79B suggests that the genetic origin of PCNSL is distinct from that of other non-CNS DLBCLs [14]. However, there is no biomarker-oriented regimen currently being carefully developed for this disease entity. Future studies will focus on how to determine the optimal targeted chemoimmunotherapy by molecular stratification. A few questions need to be answered. For example, what is the appropriate schedule for combining HD-MTX, BTKis and iMiDs in such a disease commonly occurring in elderly patients? Is it necessary to apply antifungal prophylaxis when combining BTKis and HD chemotherapy? Which targeted agent is most suitable for induction or maintenance? How can ICANS be controlled during CAR-T cell therapy? A more precise stratification system depending on age, performance status, molecular pathological findings, genetic profile and cellular bioinformation needs to be developed.

## Conclusions

In the context of HD-MTX-based chemotherapy, inclusion of rituximab during induction and thiotepa-conditioned ASCT or WBRT during consolidation are currently standard choices for ND PCNSL. With the introduction of more targeted therapy focused on BCR activation and immune microenvironment modulation, the outcome of PCNSL patients has recently improved (Fig. 1). The optimal combination of novel targeted therapies and HD-MTX backbone in frontline will build a “stat-of-art” strategy in this rare malignancy.

## Data Availability

The registered BTKi trials of CNS lymphoma were sourced from the website Clinicaltrials.gov, accessed in October 2020. The associated pharmacokinetic conditions of the agent recommended by NCCN Guideline for PCNSL were collected from the public data sources of drugbank (https://www.drugbank.ca/drugs).

## Abbreviations

a: Associated conditions collected from the public data sources of drugbank (https://www.drugbank.ca/drugs)
A, AraC: Cytarabine
ALT: Alanine aminotransferase
APTT: Activated partial thromboplastin time
ASCT: Autologous hematopoietic stem cell transplantation
BCNU: Carmustine
BCR: B-cell antigen receptor
BBB: Blood-brain barrier
BTKi: Bruton tyrosine kinase inhibitor
c: Corrected for protein binding
CAR-T: Chimeric antigen receptor T cells
con: Consolidation
CHOP: Cyclophosphamide, doxorubicin, vincristine, prednisone
CLL: Chronic lymphocytic leukemia
CNS: Central nervous system
CR: Complete remission
CRES: CAR-T cell-related encephalopathy syndrome
CRS: Cytokine release syndrome
CRu: Complete remission unconfirmed
CSF: Cerebrospinal fluid
DA-TEDDi-R: Dose adjusted etoposide, temozolomide, liposomal doxorubicin, dexamethasone, intrathecal cytarabine
DLBCL: Diffuse large B-cell lymphoma
DXM: Dexamethasone
EBV: Epstein-Barr virus
EFS: Event-free survival
F: Fotemustine
HD-MTX: High dose methotrexate
I: Ifosfamide
IBER: Ibrutinib, rituximab, ifosfamide and etoposide
ICANS: Immune effector cell-associated neurotoxicity syndrome
IgH: Immunoglobulin heavy chain
iMiD: Immunomodulatory drugs
iv: Intravenous
M, MTX: Methotrexate
MMSE: Mini Mental State Examinations
MRE: Methotrexate, rituximab, and etoposide
MV: Molecular weight
NA: Not available
NCCN: National comprehensive cancer network
NCR: No complete remission
ND: Newly diagnosed
NGR-hTNF: Tumor necrosis factor-α coupled with CNGRCG peptide
non-GCB: Non-germinal center B-cell-like type
ORR: Overall response rate
OS: Overall survival
p: Nonhuman primates
PCNSL: Primary central nervous system lymphoma
PD-1: Programmed cell death protein-1
PFS: Progression-free survival
PR: Partial remission
Pred: Prednisone
Pro: Procarbazine
PVRL: Primary vitreoretinal lymphoma
QoL: Quality of life
R: Rituximab
r/r: Refractory/relapse
RCT: Randomized control trial
R-MVP: Rituximab, methotrexate, procarbazine and vincristine
RT: Radiotherapy
SCNSL: Secondary central nervous system lymphoma
T: Thiotepa
Ten: Teniposide
TRAE: Treatment-related adverse effect
TMZ: Temozolomide
WBRT: Whole brain radiotherapy
w/w: Wait and watch
V: Vincristine
VP16: Etoposide
